# Skeletal muscle IL-6 regulates muscle substrate utilization and adipose tissue metabolism during recovery from an acute bout of exercise

**DOI:** 10.1371/journal.pone.0189301

**Published:** 2017-12-18

**Authors:** Jakob G. Knudsen, Anders Gudiksen, Lærke Bertholdt, Peter Overby, Ida Villesen, Camilla L. Schwartz, Henriette Pilegaard

**Affiliations:** Section for Cell Biology and Physiology, Department of Biology, University of Copenhagen, Copenhagen, Denmark; University of Birmingham, UNITED KINGDOM

## Abstract

An acute bout of exercise imposes a major challenge on whole-body metabolism and metabolic adjustments are needed in multiple tissues during recovery to reestablish metabolic homeostasis. It is currently unresolved how this regulation is orchestrated between tissues. This study was undertaken to clarify the role of skeletal muscle derived interleukin 6 (IL-6) in the coordination of the metabolic responses during recovery from acute exercise. Skeletal muscle specific IL-6 knockout (IL-6 MKO) and littermate Control mice were rested or ran on a treadmill for 2h. Plasma, skeletal muscle, liver and adipose tissue were obtained after 6 and 10h of recovery. Non-exercised IL-6 MKO mice had higher plasma lactate and lower plasma non-esterified fatty acids than Controls. The activity of pyruvate dehydrogenase in the active form was, in skeletal muscle, higher in IL-6 MKO mice than Controls in non-exercised mice and 6h after exercise. IL-6 MKO mice had lower glucose transporter 4 protein content in inguinal adipose tissue (WAT) than Control in non-exercised mice and 10h after treadmill running. Epididymal WAT hormone sensitive lipase phosphorylation and inguinal WAT mitogen activated kinase P38 phosphorylation were higher in IL-6 MKO than Control mice 6h after exercise. These findings indicate that skeletal muscle IL-6 may play an important role in the regulation of substrate utilization in skeletal muscle, basal and exercise-induced adaptations in adipose tissue glucose uptake and lipolysis during recovery from exercise. Together this indicates that skeletal muscle IL-6 contributes to reestablishing metabolic homeostasis during recovery from exercise by regulating WAT and skeletal muscle metabolism.

## Introduction

A single bout of exercise has profound effects on whole-body metabolism, increasing insulin sensitivity [1], fatty acid oxidation [2] and activating metabolic pathways and gene transcription in skeletal muscle, adipose tissue and liver during recovery [3–7]. However, the molecular mechanisms underlying the coordination of whole-body metabolism and tissue specific gene transcription during recovery from exercise are unclear. Circulating factors such as myokines may contribute to the regulation of these changes.

Interleukin 6 (IL-6) is a myokine, which has been shown to be released from human skeletal muscle during exercise [8] and been suggested to be implicated in the regulation of metabolism during exercise. In accordance, infusion of IL-6 in human subjects has been demonstrated to increase insulin-stimulated whole-body glucose uptake and oxidation [9] as well as exercise-induced whole-body glucose production and uptake [10]. In fed mice injection of IL-6 has been shown to reduce pyruvate dehydrogenase (PDHa) activity in skeletal muscle [11] while lack of skeletal muscle IL-6 has been demonstrated to increase skeletal muscle PDHa activity in mice [12]. Together this indicates that IL-6 is involved in decreasing carbohydrate metabolism in skeletal muscle.

Infusion of IL-6 has also been shown to increase whole-body lipolysis and fat oxidation in humans [13, 14]. In accordance, *ex vivo* incubation of rodent adipose tissue with IL-6 has been demonstrated to increase lipolysis [15]. Moreover, IL-6 infusion has been reported to increase hormone sensitive lipase (HSL) mRNA content in human adipose tissue [16] and that IL-6 whole-body knockout (KO) mice have lower 5’AMP activated protein kinase (AMPK) phosphorylation in adipose tissue during exercise than wild-type mice [17]. Together this indicates that IL-6 signaling plays a role in inducing adipose tissue lipolysis with concomitant release of fatty acids to the circulation and increased fatty acid oxidation.

A previous study has shown that whole-body ablation of IL-6 resulted in lower basal uncoupling protein 1 (UCP1) protein content in mouse inguinal adipose tissue (iWAT) and loss of exercise training-induced increases in iWAT UCP1 mRNA [18]. Although IL-6 has been demonstrated not to affect exercise-induced changes in mitochondrial content in rodent visceral adipose tissue [19] it may be suggested that IL-6 is important for the ability to uncouple ATP production in WAT in order to relieve exercise-induced metabolic stress in WAT. In addition, skeletal muscle specific IL-6 KO mice have been shown to have lower glucose transporter 4 (GLUT4) content in iWAT [20], indicating that skeletal muscle IL-6 may contribute to the regulation of carbohydrate metabolism in adipose tissue.

Therefore, it may be suggested that skeletal muscle derived IL-6 acts as an endocrine signal to orchestrate the regulation of glucose and fat metabolism both during and post exercise. Thus, the aim of this study was to test the hypothesis that knockout of skeletal muscle IL-6 alters protein phosphorylation, protein content and metabolic regulation in skeletal muscle, adipose tissue and liver during the recovery from a prolonged acute bout of exercise.

## Methods

### Animals

All experiments were approved by and performed according to the guidelines provided by Raadet for dyreforsoeg, Danish ministry of food, agriculture and fisheries. Male skeletal muscle-specific IL-6 knockout (IL-6 MKO) mice and floxed littermates (Control), were bred by crossing C57BL/6 mice carrying a loxP insert surrounding exon 2 of the *Il6* gene with mice carrying a Cre recombinase under the control of the myogenin promoter in addition to the loxP insert as previously described [20, 21]. Skeletal muscle IL-6 deletion was verified in all IL-6 MKO mice in the current study by DNA genotyping on muscle tissue. The animals were housed at 22° in a 12:12h light: dark cycle with ad libitum access to standard rodent chow (#1320, Brogaarden, Lynge, Denmark) and water.

### Acute exercise protocol

At 11 weeks of age, IL-6 MKO and Control littermate mice were placed in single housing. Before the experiment the mice were adapted to treadmill running (TSE Systems, Bad Homburg, Germany), 10 min twice a day, for five consecutive days. Following a 24h resting period the mice performed a single exercise bout on the treadmill. The exercise bout consisted of 120 min of running at 14 m/min with a 10° incline. Immediately following the exercise bout, the mice were returned to their respective cages with ad libitum access to food and water as no difference in food intake has previously been observed between IL-6 MKO and control mice. Mice remaining in their cages during the experiment served as rested controls (Non-exercised). The pre-exercised mice were euthanized by cervical dislocation either 6h (n = 10) or 10h (n = 10) after the exercise bout and the Non-exercised mice were euthanized together with the prior exercised mice with n = 5 at each time point. Quadriceps muscles were immediately removed and snap frozen in liquid nitrogen, followed by collection of trunk blood and liver as well as epididymal and inguinal adipose tissues. Plasma was generated from whole blood by centrifugation at 2600g, 4°C, 15 min and both tissue and plasma samples were stored at -80°C until further analyses.

### Plasma glucose, lactate and FFA

Plasma glucose was measured enzymatically as previously described [22]. Plasma lactate was measured from PCA extract of plasma samples in a reaction mix containing glycyl-glycine, H_2_O, NAD^+^ and glutamic acid using the same general principles as for plasma glucose. NEFA content was determined using WAKO HR Series NEFA-HR (2) (WAKO Diagnostics, Richmond, VA, USA) according to manufacturer’s guidelines.

### Plasma insulin and IL-6

Plasma insulin and IL-6 concentrations were determined using a mesoscale discovery mouse insulin kit (K152BZC-2) and V-Plex cytokine assay (K152A0H-2), respectively, according to the manufacturers guidelines (Mesoscale Discovery, Rockville, MD, USA).

### Liver and skeletal muscle glycogen content

Liver and skeletal muscle glycogen content was determined on approximately 10mg of crushed tissue by boiling in 1M HCl for 2h to convert glycogen to glycosylic units followed by enzymatic determination of glycosylic units as previously described [22].

### Skeletal muscle PDHa acticity

The activity of PDH in the active form (PDHa) was measured in homogenates from quadriceps as previously described [23–25] with modifications [26] and normalized to the relative creatine content in the homogenate as previously described [12, 27].

### RNA isolation and reverse transcription

Total RNA was isolated from crushed tissue using guanidinium thiocyanate—phenol-chloroform extraction [28] as previously described [3]. In addition, Superscript II RNase H^−^ system and Oligo dT (Invitrogen, Carlsbad, CA, USA) were used to reverse transcribe mRNA to cDNA.

### Real time PCR

The mRNA content of target genes was determined by real time PCR (ABI79000 prism (Applied Biosystems). TaqMan probes and primers (Table 1) targeting specific mRNAs were designed from mRNA sequences obtained from the ensemble database (ww.ensemble.org) using Primer Express (Applied Biosystems, Foster City, California, US). TaqMan probes were 5’ 9-6-carboxyfluorescein (FAM) and 3 ‘9-6-carboxy-N,N,N9,N9-tetramethylrhodamine (TAMRA) labeled except for the β-actin and TBP which were pre-developed assay reagents (Applied Biosystems). Samples were run in triplicates and a serial dilution of a pool of all samples was used to create a standard curve from which Ct values of samples were converted to relative amounts. Various potential endogenous controls were significantly affected by the intervention or the genotype in liver, adipose tissue and skeletal muscle. Therefore inguinal adipose tissue (iWAT) target mRNA was normalized to ssDNA concentration determined by OliGreen as previously described [29], epididymal adipose tissue (eWAT) and liver target mRNA were normalized to β-actin mRNA content and skeletal muscle target mRNA was normalized to TBP mRNA.

**Table 1:** 

Gene	Forward primer	TaqMan Probe	Reverse primer
**IL-6**	5'CTTAATTACACATGTTCTCTGGGAAA 3'	5' ATCAGAATTGCCATTGCACAACTCTTTTCTCAT 3'	5' CAAGTGCATCATCGTTGTTCATAC 3'
**SOCS3**	FP: 5' GCCACCTGGACTCCTATGAGAA 3'	5' GAGCATCATACTGATCCAGGAACTC 3'	5' TGACCCAGCTGCCTGGACCCATT 3'
**UCP1**	5' AAGCGTACCAAGCTGTGCG A 3'	5' AGAAAAGAAGCCACAAACCCTT 3'	5' CCATGTACACCAAGGAAGGACCGACG 3'
**G6Pase**	5' TTCAACCTCGTCTTCAAGTGGAT 3',	5' TGTTTGGACAACGCCCGTATTGGTG 3'	5' CACGGAGCTGTTGCTGTAGTAGTC 3'
**PEPCK**	5' GGAAGAGGACTTTGAGAAAGCAT 3'	5' CCAGGTTCCCAGGGTGCATGAAAG 3'	5' TCAGTTCAATACCAATCTTGGCC 3'

Real time PCR primer and TaqMan probe sequences.

### Protein lysate preparation

Lysate generation was performed on 30-80mg of tissue depending on tissue after homogenization (Tissue Lyzer, Qiagen, Germany) in 10% glycerol, 20 mM Na-pyrophosphate, 150 mM NaCl, 50 mM hepes, 1% NP-40, 20 mM b-glycerolphosphate, 10 mM NaF, 1 mM EDTA, 1 mM EGTA, 20 μg/ml Aprotinin, 10 μg/ml leupeptin, 2 mM Na3VO4 and 3 mM benzamidine adjusted to pH 7.4. After homogenization liver and skeletal muscle samples were left end over end for 60min, while SDS was added to adipose tissue samples to a final concentration of 2% as previously described [20]. Samples were then centrifuged at 16000g, 4°C for 20min and the supernatant was transferred to new tubes. Lysate protein concentration was determined using a bicinchoninic acid assay and samples were adjusted to concentrations of 0.5 (eWAT), 1 (skeletal muscle and iWAT) and 2 μg/μl (liver) in sample buffer containing SDS.

### SDS page and western blotting

Samples were separated by SDS-Page on hand casted gels and blotted to a PVDF membrane (Millipore, Hellerup, Denmark) blocked in 3% fish gel (Sigma Aldrich) in Tris buffered saline containing 0.05% tween 20. Membranes were then incubated in primary antibody recognizing ACC^Ser212/79^phosphorylation (#07–303, Millipore, Darmstad, Germany), AMPK^Thr172^ phosphorylation (#2535S, Cell Signaling, Danvers, MA, USA), AMPKα2 (Hardie, G,), GAPDH (Cell Signaling, Danvers, MA, USA), G6Pase (#sc-27198 (c14), Santa Cruz Biotechnology, Dallas TX, USA), GLUT4 (#PAI-1065, ABR), HSL^Ser660^and ^Ser565^ phosphorylation as well as HSL protein (#8334 and #8334 Cell Signaling, respectively), HKII (#2867, Cell Signaling), LDHa (#sc-27230 Santa Cruz Biotechnology), P38^Thr180/Tyr182^ phosphorylation (#4511 Cell Signaling), P38 (#9212, Cell Signaling), PDH^Ser300^ and ^Ser295^ phosphorylation (Hardie, G), PDH^Ser232^ phosphorylation (#AP1063, Calbiochem, Millipore), PDH-E1α (Hardie, G), PDK4 (Hardie, G), PEPCK (#10004943, Cayman chemicals, Elsworth, MI, USA), or UCP1 (#AB10983, Abcam, Cambridge, UK) overnight. The following day the membranes were incubated with HRP-conjugated secondary antibody (Dako, Glostrup, Denmark) and bands were visualized using luminescence on a LAS4000 and quantified using Image quant 8.1 (GE Health care, Freiburg, Germany). ACC1 and 2 protein content was determined using HRP-conjugated Streptavidin (Dako, Glostrup, Denmark),

### Statistics

Results are presented as mean ± standard error. A two-way ANOVA was applied to determine the effect of acute exercise and genotype on mRNA content, protein content, phosphorylation, and plasma levels, and a Student–Newman–Keuls post hoc test was used to locate differences between genotypes and recovery time points. For liver SOCS3 mRNA content equal variances were not present in the two way ANOVA test and therefore a one-way ANOVA was applied to determine the effect of acute exercise within each genotype separately. A value of p<0.05 was considered significant. Furthermore, to convey potentially important information and reduce the risk of committing type II errors, tendencies, defined as 0.05≤p< 0.1, were reported in the results section but otherwise not included in tables or figures. However these statistical results should be interpreted with care as they may also be prone to type I errors.

## Results

### Plasma

**Fig 1:**
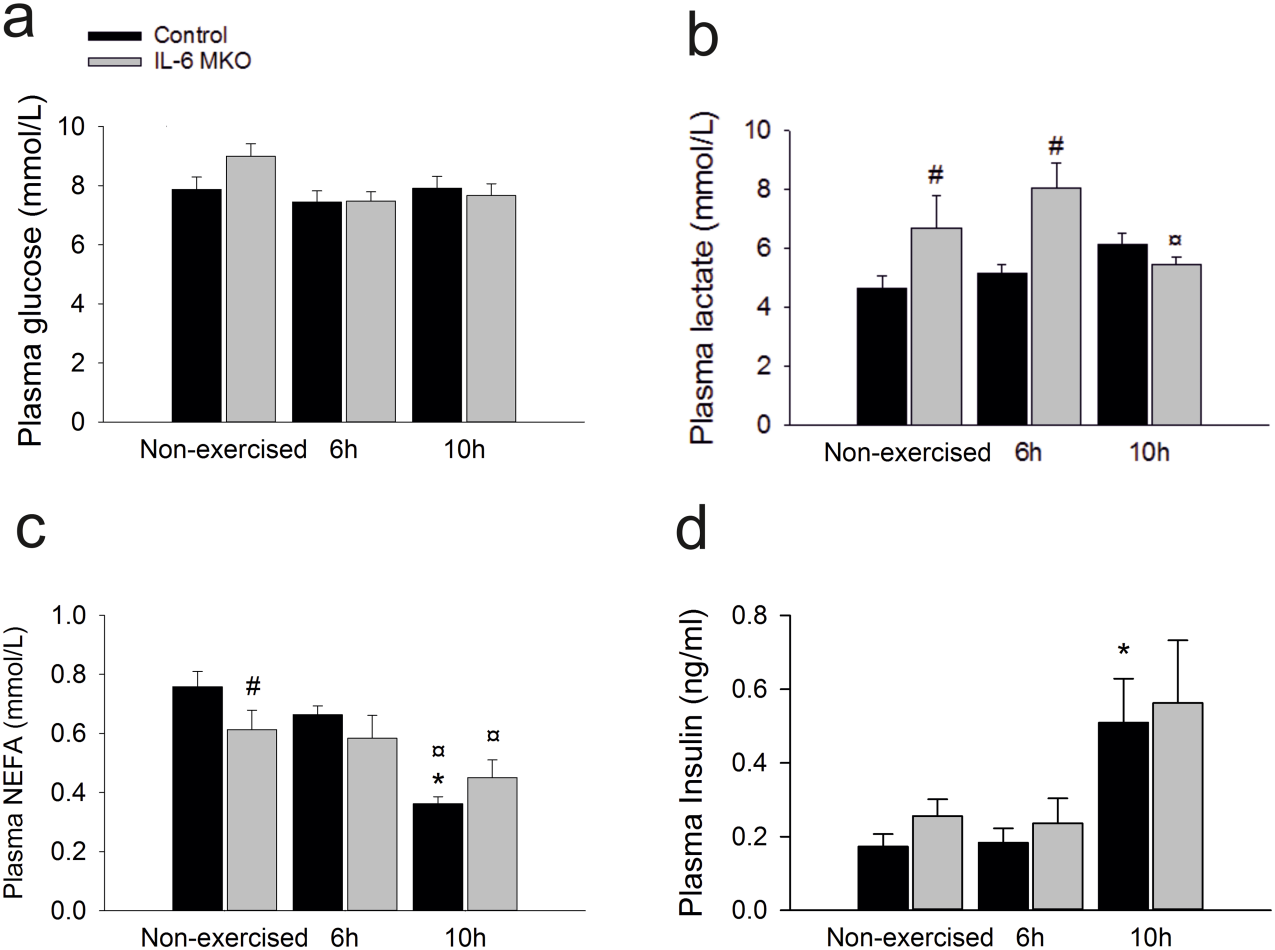
Plasma concentrations of glucose (a), lactate (b), NEFA (c) and insulin (d) in non-exercised mice as well as 6h and 10h after 2h of treadmill running in Controls and IL-6 MKO mice. Values are mean ± SE (n = 9–10) *: significantly different from non-exercised mice within given genotype (p<0.05), ¤: significantly different from 6h within given genotype (p<0.05), #: significantly different from Control within given time point (p<0.05).

Regulation of plasma substrate levels and endocrine factors during recovery from exercise influences the metabolic responses in skeletal muscle, liver and adipose tissue. Therefore, the plasma concentrations of IL-6, non-esterified fatty acids (NEFA), lactate, glucose and insulin were measured. Plasma glucose concentration tended to be lower (p = 0.066) at 6 and 10h of recovery than in non-exercised mice in both genotypes (Fig 1a). While changes in plasma glucose were limited in response to exercise and lack of skeletal muscle IL-6, plasma lactate concentrations were higher (p<0.05) in IL-6 skeletal muscle specific KO (MKO)mice than Controls in both non-exercised mice and 6h after exercise. Furthermore, plasma lactate in IL-6 MKO mice was lower (p<0.05) 10h after exercise than at 6h, but unchanged in Control mice (Fig 1b). In addition, plasma NEFA concentration was lower (P<0.05) in IL-6 MKO mice than Controls within non-exercised mice. The two genotypes had similar non-esterified free fatty acid (NEFA) concentrations after exercise as the plasma NEFA concentrations were lower (P<0.05) 10h after exercise than at 6h after exercise in both Control and IL6 MKO mice. However, in controls plasma NEFA was also lower (P<0.05) 10h after exercise than in non-exercised mice (Fig 1c). There were no differences in the plasma insulin concentrations between 6h of recovery and non-exercised mice in either genotype, but the plasma insulin concentrations were higher (p<0.05) 10h after exercise than in non-exercised mice within Controls, but not IL-6 MKO mice (Fig 1d). Intriguingly, these changes were observed without significant changes in the plasma concentrations of IL-6 (Table 2).

**Table 2:** 

	Non-exercised		6h		10h	
	Control	IL-6 MKO	Control	IL-6 MKO	Control	IL-6 MKO
**Plasma IL-6 (pg/ml)**	5.5±1.3	3.5±0.1	20.0±8.8	12.5±3.2	5.1±1.0	2.2±0.4
SkM IL-6 mRNA/TBP mRNA (AU)	1.1±0.2	1.2±0.2	1.5±0.2	0.6±0.2#	1.8±0.2	1.2±0.2#
SkM SOCS3 mRNA/TBP mRNA (AU)	1.1±0.1	1.2±0.1	1.1±0.2	1.2±0.1	1.4±0.2	1.7±0.3

Plasma levels of IL-6 and Skeletal muscle IL-6 and SOCS3 mRNA content in non-exercised mice as well as 6h and 10h after 2h of treadmill running in Control and IL-6 MKO mice. SkM mRNA is given in arbitrary units (AU). Values are mean ± SE (n = 9–10)

*: significantly different from non-exercised mice within given genotype (p<0.05),

^#^: significantly different from Control within given time point (p<0.05).

### Adipose tissue

**Fig 2:**
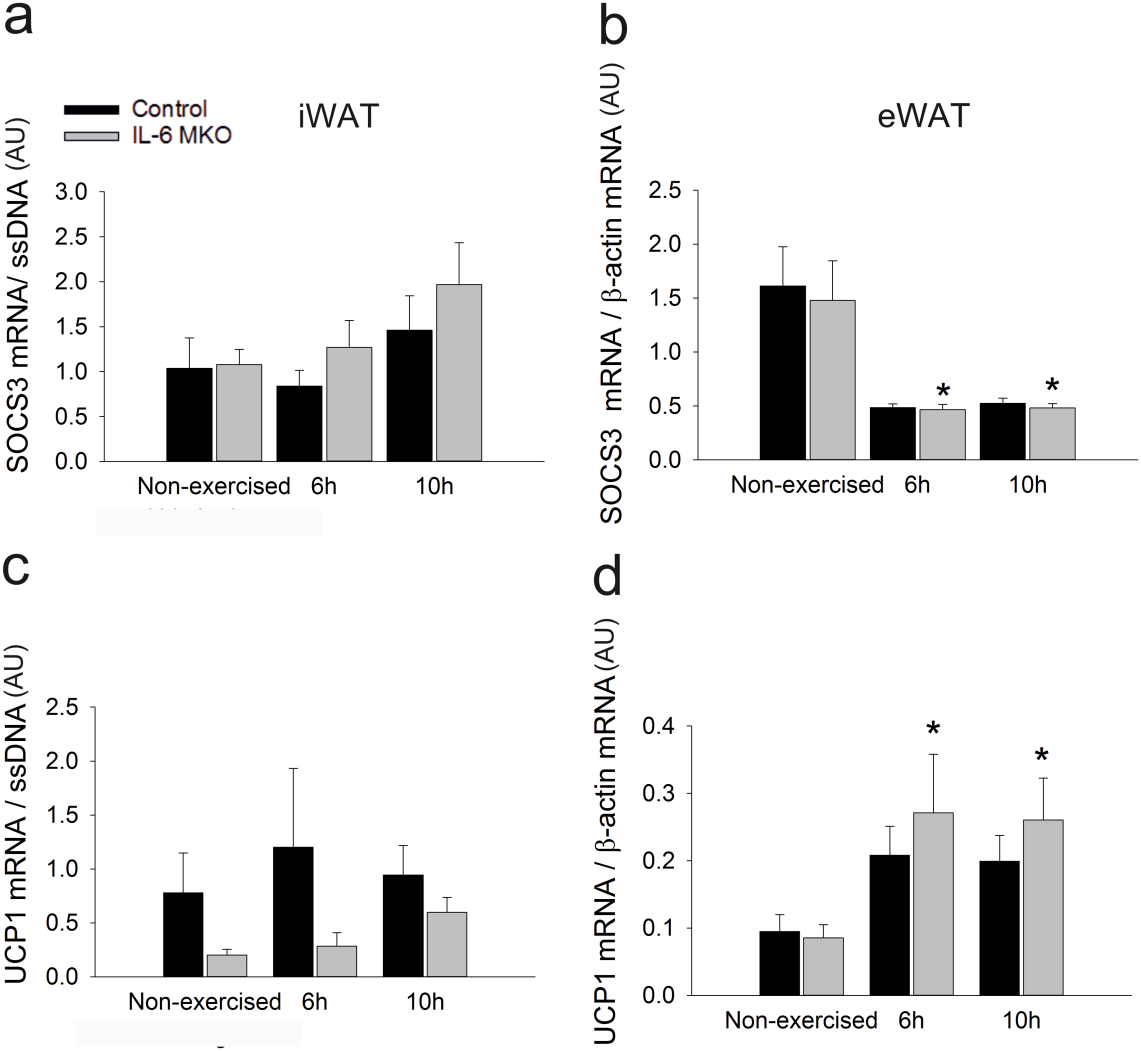
iWAT SOCS3 mRNA content (a) eWAT SOCS3 mRNA content (b) iWAT UCP1 mRNA content (c) eWAT UCP1 mRNA content (d) in non-exercised mice as well as 6h and 10h after 2h of treadmill running in Control and IL-6 MKO mice. Values are mean ± SE; (n = 9–10). *: significantly different from non-exercised within given genotype (p<0.05), ¤: significantly different from 6h within given genotype (p<0.05), #: significantly different from Control within given time point (p<0.05).

It is unknown whether skeletal muscle-derived IL-6 plays a role in the regulation of metabolic and adaptive responses in adipose tissue during recovery from exercise. Therefore, metabolic regulation and adaptive responses were evaluated in inguinal white adipose tissue (iWAT) and epididymal WAT (eWAT). Firstly, the canonical IL-6 signaling pathway was investigated. Suppressor of cytokine signalling 3 (SOCS3) mRNA content in iWAT tended to be higher (P = 0.057) 10h after exercise than in non-exercised mice in both genotypes (Fig 2a). Contrarily, in eWAT SOCS3 mRNA was lower (P<0.05) at both 6 and 10h after exercise than in non-exercised in both Control and IL-6 MKO mice (Fig 2b). These findings indicate that there was no direct effect of lack of skeletal muscle IL-6 on canonical IL-6 signaling in adipose tissue.Furthermore, in iWAT, the Uncoupling protein (UCP)1 mRNA content tended to be lower (p = 0.057) in IL-6 MKO mice than in Control mice (Fig 2c). The UCP1 mRNA content in eWAT was in IL-6 MKO mice higher (p<0.05) 6h and 10h after exercise than in non-exercised mice, while exercise had no effect on eWAT UCP1 mRNA in Control mice (Fig 2d).

**Fig 3:**
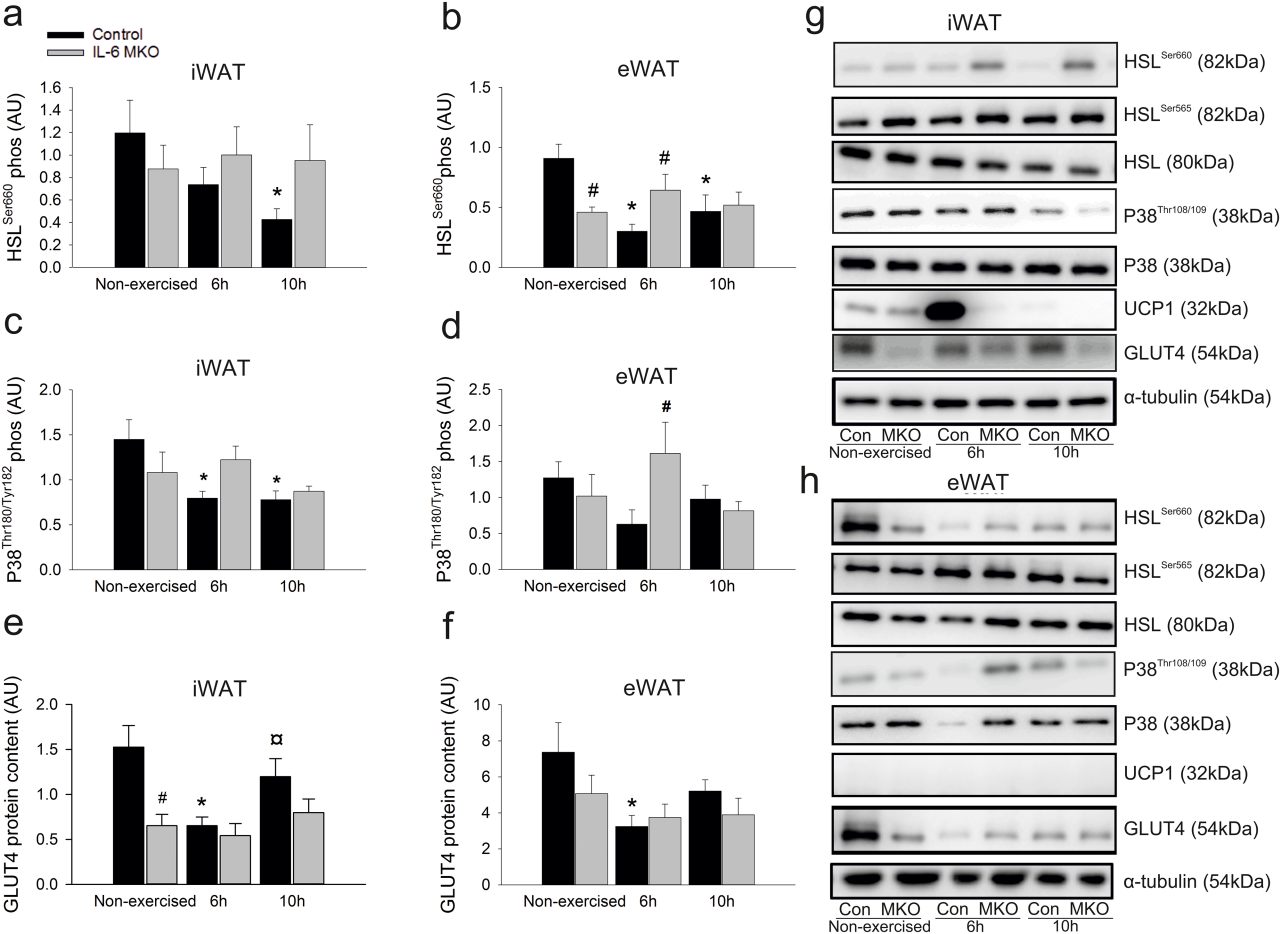
iWAT HSL^Ser660^ phosphorylation (a), eWAT HSL^Ser660^ phosphorylation (b), iWAT P38^Tyr180/182^ phosphorylation (c), eWAT P38^Tyr180/182^ phosphorylation (d), iWAT GLUT4 protein content (e), iWAT GLUT4 protein content (f), iWAT (g) and eWAT in non-exercised mice as well as 6h or 10h after 2h of treadmill running in d IL-6 MKO and Control mice. (h) Representative blots of HSL^565^ phosphorylation, HSL, UCP1 and P38 protein content. Values are mean ± SE; (n = 9–10). *: significantly different from non-exercised within given genotype (p<0.05), ¤: significantly different from 6h within given genotype (p<0.05), #: significantly different from Control within given time point (p<0.05).

To estimate signaling events involved in lipolytic activity, phosphorylation of hormone sensitive lipase (HSL), a key enzyme in lipolysis, was investigated. In Control mice, phosphorylation of the activating HSL^Ser660^ site in iWAT was lower (p<0.05) 10h after exercise than in non-exercised mice (Fig 3a and 3b). However, there was no change in HSL^Ser660^ phosphorylation in iWAT in response to exercise in IL-6 MKO mice (Fig 3a and 3b). Similarly, HSL^Ser660^ phosphorylation in eWAT was lower (p<0.05) both at 6h and 10h in exercised than in non-exercised mice within Controls, while there were no changes in HSL^Ser660^ phosphorylation in eWAT in response to exercise in IL-6 MKO mice. Moreover, the difference between the two genotypes was already apparent in the non-exercised group, where HSL^Ser660^ phosphorylation was lower (p<0.05) in IL-6 MKO than in Control mice (Fig 3b) and 6h after exercise, where HSL^Ser660^ phosphorylation was higher (p<0.05) in IL-6 MKO than Control mice. There was no effect of exercise or genotype on the inactivating HSL^Ser565^ phosphorylation site in iWAT, but in eWAT HSL^Ser565^ phosphorylation was higher (p<0.05) 10h after exercise than in non-exercised controls (Table 3 and Fig 3g and 3h). Furthermore, in iWAT, HSL protein content was lower (p<0.05) 10h after exercise than in non-exercised mice in Controls and there was an overall tendency for lower (p = 0.08) iWAT HSL protein content in IL-6 MKO than Control mice. (Table 3, Fig 3g and 3h).

**Table 3:** 

	Non-exercised		6h		10h	
iWAT	Control	IL-6 MKO	Control	IL-6 MKO	Control	IL-6 MKO
HSL^Ser565^	0.77(±0.06)	0.62(±0.08)	0.74(±0.07)	0.73(±0.11)	0.82(±0.13)	0.77(±0.06)
HSL	1.04(±0.08)	0.86(±0.1)	0.98(±0.08)	0.81(±0.06)	0.76(±0.09)*	0.79(±0.04)
P38	0.87(±0.05)	0.81(±0.06)	0.79(±0.07)	0.70(±0.07)	0.88(±0.04)	0.74(±0.04)
UCP1	1.18(±0.66)	1.03(±0.47)	2.15(±1.16)	0.35(±0.15)	0.68(±0.22)	0.92(±0.37)
**eWAT**						
HSL^Ser565^	0.62(±0.07)	0.74(±0.13)	0.55(±0.05)	0.72(±0.12)	0.82*(±0.09)	0.73(±0.1)
HSL	2.74(±0.43)	2.78(±0.5)	2.24(±0.32)	2.85(±0.55)	2.81(±0.34)	2.62(±0.53)
P38	0.76(±0.12)	0.77(±0.07)	0.40(±0.05)*	0.56(±0.1)	0.50(±0.05)	0.50(±0.05)*
UCP1	**-**	**-**	**-**	**-**	**-**	**-**

Data for results shown only as representative blots. The table shows protein content in iWAT, eWAT, in non-exercised mice as well as 6h or 10h after 2h of treadmill running in d IL-6 MKO and Control mice. Values are mean ± SE; (n = 9–10).

*: significantly different from non-exercised within given genotype (p<0.05).

P38 map-kinase signaling was determined in iWAT and eWAT. P38^Thr180/Tyr182^ phosphorylation in iWAT was lower (p<0.05) in Control mice 6 and 10h after exercise than in non-exercised mice, but unchanged after exercise in IL-6 MKO mice. Furthermore, the phosphorylation of P38^Thr180/Tyr182^ in iWAT tended to be lower (p = 0.098) in IL-6 MKO than in Controls within non-exercised mice and to be higher (p = 0.062) in IL-6 MKO than Control mice 6h after exercise (Fig 3c). Although P38^Thr180/Tyr182^ phosphorylation in iWAT was unchanged after exercise in both Control and IL-6 MKO mice (Fig 3d), P38^Thr180/Tyr182^ phosphorylation in eWAT was higher (p<0.05) in IL-6 MKO than Control mice 6h after exercise (Fig 3d). In addition iWAT p38 protein content was lower (P<0.05) 6h after exercise and tended to be lower (P = 0.09) 10h after exercise in Control mice than in non-exercised mice. Similarly, p38 protein content was lower (P<0.05) at 10h of recovery and tended to be lower (P = 0.052) 6h after exercise in IL-6 MKO mice than in non-exercised (Table 3 and Fig 3g).

To investigate whether skeletal muscle-derived IL-6 plays a role in exercise-induced Glucose transporter (GLUT) 4 regulation in adipose tissue, GLUT4 protein content was determined in WAT. iWAT GLUT4 protein content was lower (p<0.05) 6h after exercise in Control mice than in non-exercised mice and again higher (p<0.05) 10h after exercise than 6h after exercise (Fig 3c). However, in IL-6 MKO mice iWAT GLUT4 protein content was unaltered by exercise (Fig 3e). Therefore, when IL-6 MKO and Control mice were compared, iWAT GLUT4 protein content was lower (p<0.05) in IL-6 MKO than in Controls within non-exercised mice. In addition iWAT GLUT4 protein content also tended to be lower (p = 0.095) in IL-6 MKO mice than in Control mice 10h after exercise (Fig 3e). This suggests that IL-6 MKO mice may not be able to regulate GLUT4 expression in iWAT in response to exercise. The effects of exercise and genotype on GLUT4 protein in eWAT was not nearly as pronounced as in iWAT, but GLUT4 protein content was lower (p<0.05) 6h after exercise in Controls than in non-exercised mice and unaltered in IL-6 MKO mice (Fig 3f).

### Skeletal muscle

**Fig 4:**
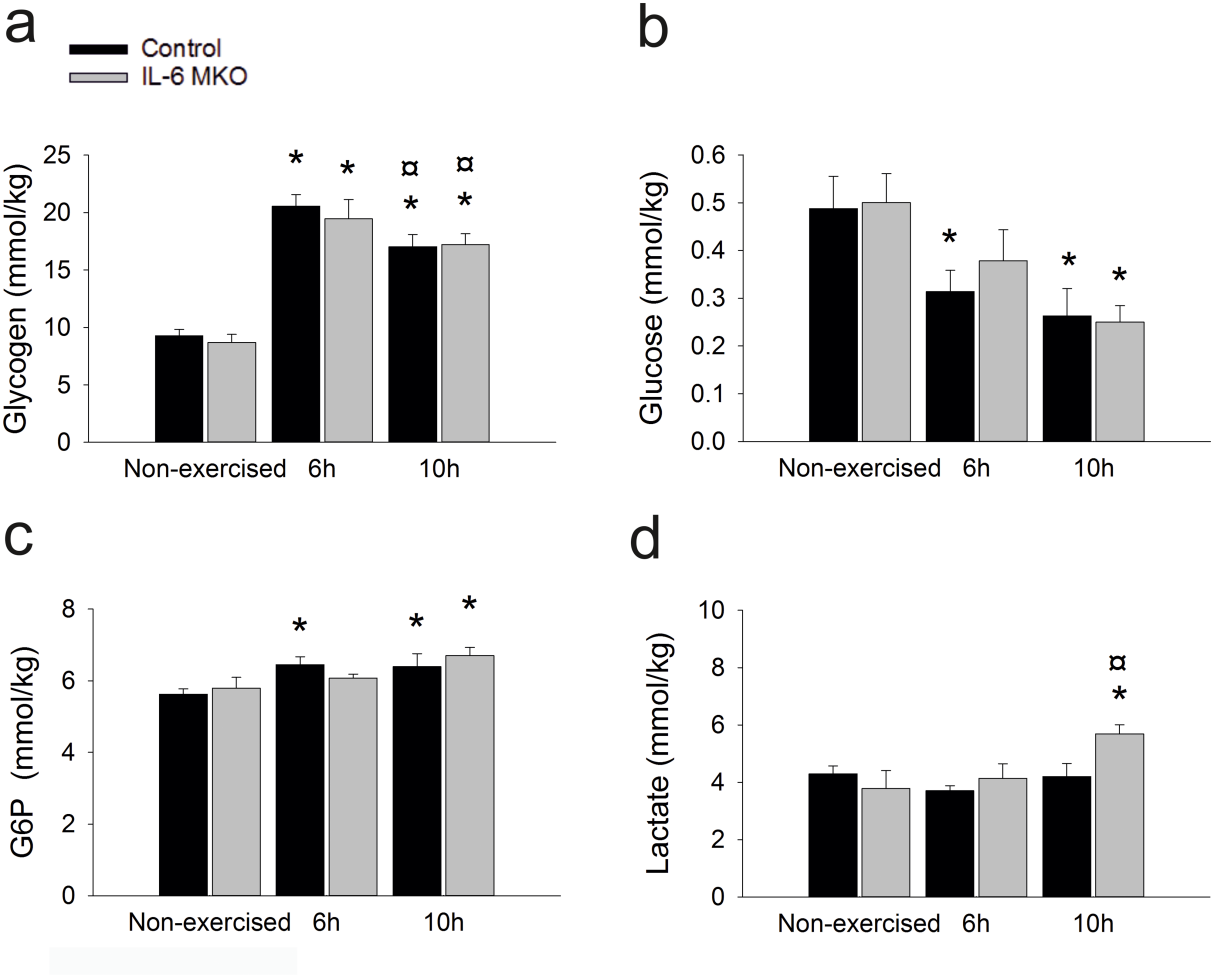
Skeletal muscle glycogen concentration (a), glucose concentration (b), glucose 6 phosphate (G6P) concentration (c) and lactate concentration (d) in non-exercised mice as well as 6h or 10h after 2h of treadmill running in Control and IL-6 MKO mice. Values are mean ± SE; (n = 9–10). *: significantly different from non-exercised within given genotype (p<0.05), ¤: significantly different from 6h within given genotype (p<0.05).

To investigate the influence of skeletal muscle IL-6 on carbohydrate metabolism in skeletal muscle during recovery from acute exercise, skeletal muscle glycogen, lactate and glucose-6-phosphate (G6P) were determined. Skeletal muscle glycogen content was higher (p<0.05) at 6 and 10h after exercise than in non-exercised mice in both genotypes. However, in Control mice glycogen was lower (p<0.05) at 10h after exercise than 6h after exercise (Fig 4a). The content of glucose in skeletal muscle was lower (p<0.05) 6h and 10h after exercise in Control mice than in non-exercised mice and lower (p<0.05) 10h after exercise in IL-6 MKO than in non-exercised mice (Fig 4b). Contrary to the glucose content, skeletal muscle G6P was higher (P<0.05) 6 and 10h after exercise in Control mice than in non-exercised mice and in IL-6 MKO mice higher (P<0.05) 10h after exercise while tending to be higher (p = 0.061) 6h after exercise than in non-exercised mice (Fig 4c). This suggests that lack of skeletal muscle IL-6 led to minor changes in carbohydrate metabolism. However, skeletal muscle lactate content was higher (P<0.05) in IL-6 MKO mice 10h after exercise than both non-exercised mice and 6h after exercise, while there was no change in skeletal muscle lactate content in Control mice (Fig 4d).

**Fig 5:**
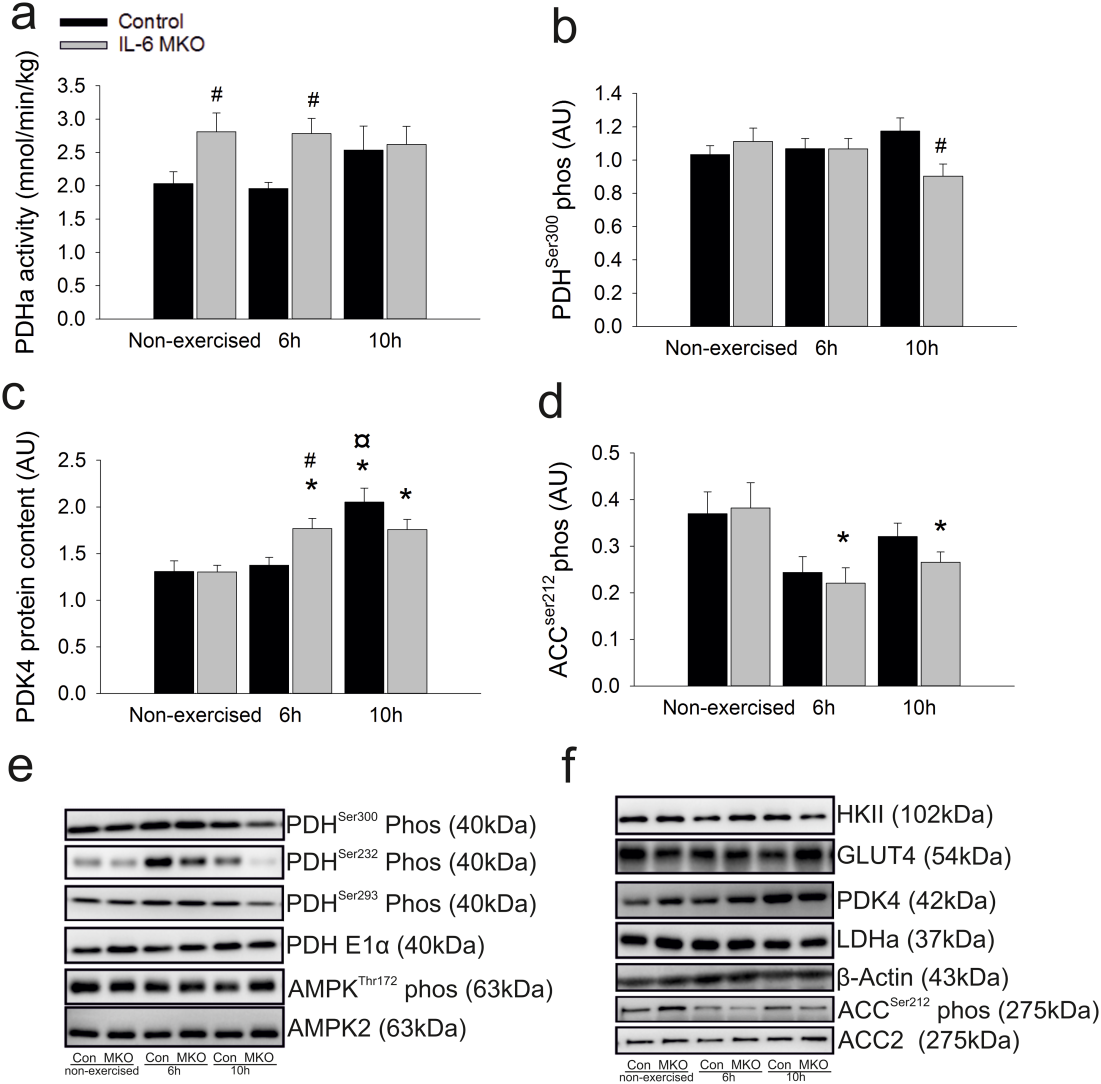
PDHa activity (a), PDH^Ser300^ phosphorylation (b) PDK4 protein content (c), ACC^Ser212^ phosphorylation (d) protein content representative blots of, PDH^Ser232^ phosphorylation, PDH^Ser293^ phosphorylation, PDH E1α, AMPK^Thr172^ phosphorylation, AMPK (e) and HKII, GLUT4, LDHa protein content and ACC2 protein content (f). in non-exercised mice as well as 6h or 10h after 2h of treadmill running in IL-6 MKO and Control mice. Values are mean ± SE;(n = 9–10). *: significantly different from non-exercised within given genotype (p<0.05), ¤: significantly different from 6h within given genotype (p<0.05), #: significantly different from Control within given time point (p<0.05).

Skeletal muscle pyruvate dehydrogenase in the active form (PDH)a activity was higher (P<0.05) in IL-6 MKO than Control mice both in non-exercised mice and 6h after exercise (Fig 5a). However, no differences were observed in PDH^Ser300^ or ^Ser293^ phosphorylation, except for a lower (p<0.05) absolute phosphorylation in IL-6 MKO than Control mice at 10h of recovery (Fig 5b). In addition, the phosphorylation of PDH^Ser232^ tended to be higher (p = 0.073) in Control mice 6h after exercise than in non-exercised and was in IL-6 MKO mice lower (P<0.05) 10h after than 6h after exercise (Table 4). Skeletal muscle PDK4 protein content was higher (p<0.05) in IL-6 MKO mice both at 6h and 10h after exercise than in non-exercised mice. However, in Control mice PDH kinase (PDK)4 protein content was higher (p<0.05) 10h after exercise than in non-exercised mice. This resulted in a genotype difference in PDK4 protein content (p<0.05) 6h after exercise (Fig 5c). In addition, no differences were observed in PDH E1α, lactate dehydrogenase (LDH)a, GLUT4 and Hexokinase (HK)II protein content (Table 3 and Fig 5).

**Table 4:** 

	Non-exercised		6h		10h	
SkM	Control	IL-6 MKO	Control	IL-6 MKO	Control	IL-6 MKO
AMPK^Thr172^	0.86(±0.06)	0.82(±0.05)	0.70(±0.05)*	0.63(±0.06)	0.71(±0.03)*	0.81(±0.06)
AMPKα2	0.95(±0.03)	0.91(±0.04)	0.88(±0.03)	0.90(±0.04)	0.86(±0.04)	0.90(±0.03)
ACC2	0.67(±0.06)	0.73(±0.07)	0.63(±0.09)	0.60(±0.06)	0.67(±0.05)	0.66(±0.03)
PDH^Ser232^	1.30(±0.33)	1.60(±0.53)	1.93(±0.35)	1.99(±0.53)	1.37(±0.27)	0.78(±0.26)
PDH^Ser293^	1.22(±0.08)	1.30(±0.08)	1.18(±0.07)	1.19(±0.06)	1.37(±0.10)	1.03(±0.1)
PDH E1α	1.04(±0.05)	1.03(±0.08)	0.96(±0.04)	0.99±(0.06)	1.11(±0.04)	0.99±(0.07)
LDH	1.06(±0.08)	1.09(±0.07)	1.16(±0.07)	1.10(±0.09)	1.11(±0.05)	1.06(±0.03)
GLUT4	1.07(±0.03)	1.05(±0.13)	1.05(±0.06)	1.02(±0.05)	1.05(±0.05)	1.00(±0.03)
HKII	1.50(±0.13)	1.54(±0.18)	1.41(±0.14)	1.36(±0.11)	1.91(±0.13)	1.41(±0.14)

Data for results shown only as representative blots. The table shows protein content in skeletal muscle (SkM) in non-exercised mice as well as 6h or 10h after 2h of treadmill running in d IL-6 MKO and Control mice. Values are mean ± SE; (n = 9–10).

*: significantly different from non-exercised within given genotype (p<0.05).

As carbohydrate metabolism seemed to be affected by a lack of skeletal muscle IL-6, it could be speculated that these changes had led to alterations in lipid metabolism. Therefore, key regulators of skeletal muscle fat oxidation were investigated. Skeletal muscle Acetyl-coenzyme A carboxylase (ACC)2^Ser212^ phosphorylation tended to be lower (p = 0.059) and was lower (p<0.05) 6h after exercise in Control and IL-6 MKO mice, respectively, and remained lower (p<0.05) 10h after exercise than in non-exercised within IL-6 MKO mice (Table 4). 5’AMP regulated protein kinase (AMPK)^Thr172^ phosphorylation was lower (p<0.05) in Control mice at 6h and 10h after exercise than in non-exercised mice. The phosphorylation of AMPK tended to be higher (p = 0.083) 10h after than 6h after exercise in IL-6 MKO mice (Table 4). In addition, There were no changes in ACC2 protein (Table 4)

### Liver

**Fig 6:**
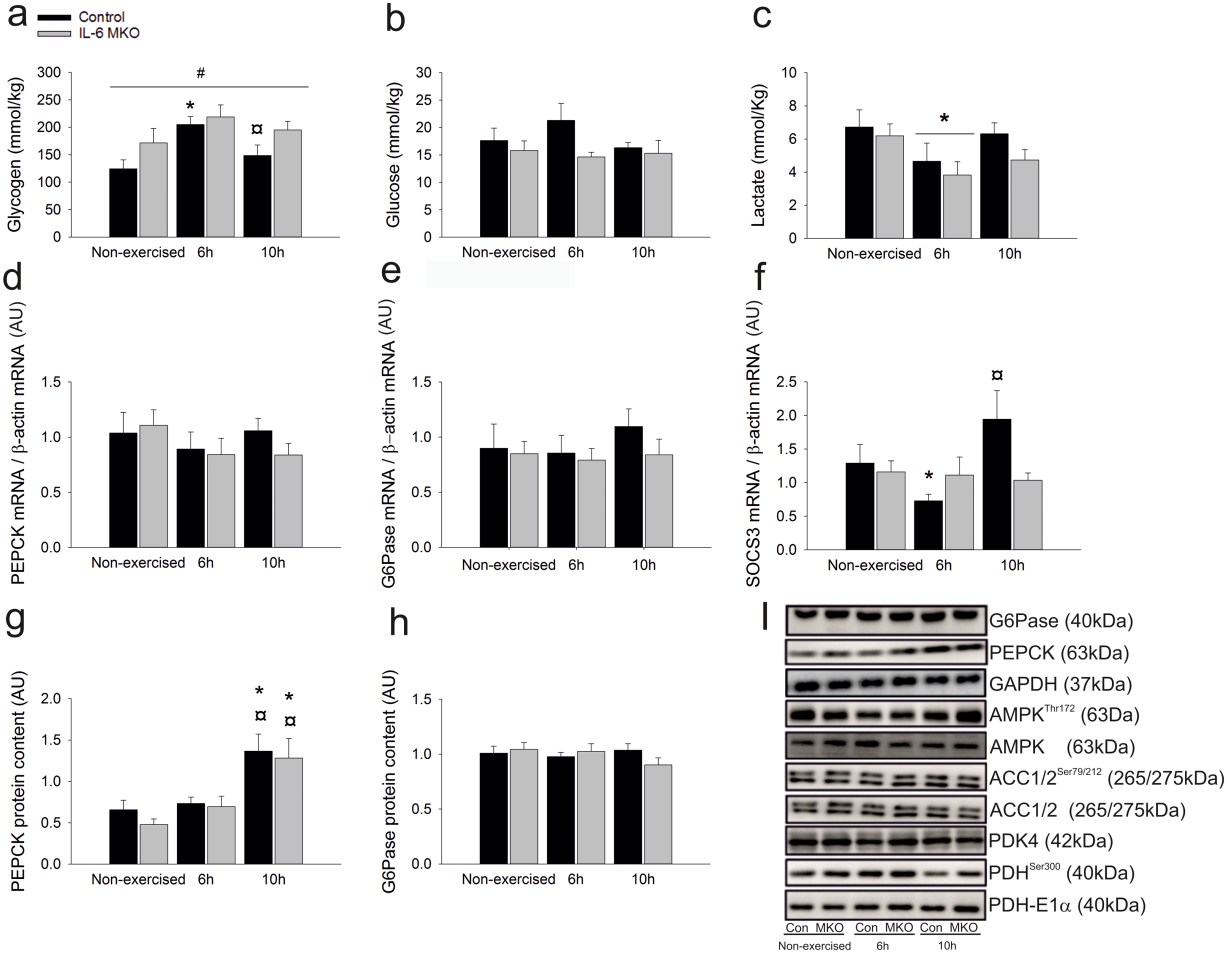
Hepatic glycogen content (a), glucose content (b), lactate content (c) PEPCK mRNA content (d), G6Pase mRNA content (e), SOCS3 mRNA content (f) PEPCK Protein content (g), G6pase protein content (h) representative blots of AMPK^Thr172^ phosphorylation, AMPK protein content, ACC1/2 ^Ser79/212^ phosphorylation, ACC1/2 protein content PDH^Ser300^ phosphorylation, PDH E1α protein content (i) in non-exercised mice as well as 6h or 10h after 2h of treadmill running in Control and IL-6 MKO mice. Values are mean ± SE; (n = 9–10) *: significantly different from rest within given genotype (p<0.05), ¤: significantly different from 6h within given genotype (p<0.05), #: significantly different from Control within given time point (p<0.05).

The impact of muscle IL-6 on hepatic carbohydrate metabolism was investigated by determining liver glycogen, glucose and lactate concentrations as well as the mRNA and protein content of gluconeogenic enzymes was determined in the liver. Liver glycogen content was higher (p<0.05) in Control mice 6h after exercise and lower (P<0.05) 10h after exercise than in non-exercised mice (p<0.05), but unaltered in IL-6 MKO mice after exercise. This led to an overall difference (p<0.05) in hepatic glycogen content between IL-6 MKO and Control mice (Fig 6a). Hepatic glucose content was unaltered after exercise in both genotypes, while hepatic lactate content was lower (p<0.05) 6h after exercise than in non-exercised mice in both genotypes (Fig 6b and 6c). In addition, hepatic phosphoenolpyruvate carboxykinase (PEPCK) and Glucose 6 phosphatase (G6Pase) mRNA content was unaltered during recovery from exercise (Fig 6c and 6d), but PEPCK protein content in the liver was higher (p<0.05) 10h after exercise than in non-exercised mice in both genotypes (Fig 6h). There were no differences in G6Pase protein content with exercise or genotype (Fig 6g).

To examine the effect of exercise on hepatic IL-6 signaling late in recovery from exercise and investigate whether canonical IL-6 signaling was reduced in the IL-6MKO mice, liver SOCS3 mRNA content was measured. In Control mice liver SOCS3 mRNA content was lower (p<0.05) 6h after exercise than in non-exercised mice, and tended to be higher (p<0.05) 10h after exercise than 6h after in non-exercised mice. There were no changes in hepatic SOCS mRNA in IL-6 MKO mice (Fig 6f).

Neither exercise nor genotype had any effect on hepatic AMPK^Thr172^, ACC^Ser212^, ACC^Ser79^ phosphorylation or ACC1 and AMPK protein content. However, non-exercised IL-6 MKO mice had higher (p<0.05) hepatic ACC2 protein content than non-exercised Control mice (Table 5 and Fig 6i). In addition, PDH^Ser300^ phosphorylation was (p<0.05) and tended (p = 0.07) to be lower 10h after exercise in Control and IL-6MKO respectively. However, neither PDK4 nor PDH-E1α protein content were affected by exercise or genotype (Table 5 and Fig 6i).

**Table 5:** 

	Non-exercised		6h		10h	
Liver	Control	IL-6 MKO	Control	IL-6 MKO	Control	IL-6 MKO
AMPK^Thr172^	0.72(±0.09)	0.62(±0.05)	0.71(±0.07)	0.56(±0.05)	0.65(±0.07)	0.61(±0.08)
AMPK	0.98(±0.22)	0.76(±0.10)	0.72(±0.10)	0.60(±0.07)	0.57(±0.06)	0.57(±0.09)
ACC1^Ser79^	0.94(±0.06)	0.86(±0.04)	0.87(±0.03)	0.95(±0.04)	0.94(±0.03)	0.83(±0.03)
ACC^Ser212^	1.15(±0.04)	1.25(±0.05)	1.21(±0.05)	1.32(±0.06)	1.30(±0.06)	1.17(±0.06)
ACC1	0.90(±0.08)	0.83(±0.08)	0.86(±0.05)	1.02(±0.07)	1.09(±0.09)	0.94(±0.11)
ACC2	0.73(±0.07)	0.97(±0.1)	0.91(±0.06)	1.00(±0.15)	0.90(±0.06)	0.82(±0.11)
PDK4	1.10(±0.21)	0.87(±0.97)	0.96(±0.14)	1.42(±0.27)	0.81(±0.14)	0.69(±0.13)
PDH^Ser300^	1.52(±0.15)	1.36(±0.15)	1.44(±0.16)	1.66(±0.11)	1.07(±0.16)*	1.08(±0.07)(*)
PDH	1.07(±0.12)	0.93(±0.14)	0.94(±0.14)	0.92(±0.08)	1.13(±0.06)	0.97(±0.09)
AMPK^Thr172^	0.72(±0.09)	0.62(±0.05)	0.71(±0.07)	0.56(±0.05)	0.65(±0.07)	0.61(±0.08)

Data for results shown only as representative blots. The table shows protein content in liver in non-exercised mice as well as 6h or 10h after 2h of treadmill running in d IL-6 MKO and Control mice. Values are mean ± SE; (n = 9–10).

*: significantly different from non-exercised within given genotype (p<0.05).

## Discussion

The major findings of this study are that an acute bout of exercise increased iWAT Glut4 protein in Control, but not IL-6 MKO mice, and that P38^Thr180/Tyr182^ phosphorylation was higher in both adipose tissues in IL-6 MKO than Control mice 6h after exercise. This suggests that lack of skeletal muscle IL-6 reduced the ability to increase iWAT GLUT4 protein after exercise and resulted in increased stress kinase signaling in adipose tissue late in recovery from prolonged exercise. In addition, the results confirm that knockout of skeletal muscle IL-6 can increase PDHa activity in skeletal muscle and supports the hypothesis that skeletal muscle IL-6 may play a role in the regulation of carbohydrate and fat metabolism late in recovery from exercise. However, muscle IL-6 does not seem to be required for the regulation of hepatic gluconeogenic capacity late in recovery from exercise.

Several studies have shown that IL-6 affects adipose tissue metabolism and lipolysis [14, 17, 30, 31]. Accordingly, the lower plasma NEFA concentrations in IL-6 MKO than Controls in the non-exercised mice indicates that lack of skeletal muscle IL-6 reduced lipolysis in adipose tissue or increased fatty acid oxidation in skeletal muscle. The findings that eWAT HSL^Ser660^ phosphorylation was lower in IL-6 MKO than Control mice suggests that muscle IL-6 influences plasma NEFA through effects on adipose tissue lipolysis. This, together with the observation that skeletal muscle PDHa activity was higher in IL-6 MKO than in Control mice and that skeletal muscle ACC phosphorylation was unaltered, strongly indicates that the change in plasma NEFA concentration was a result of decreased lipolysis in adipose tissue rather than increased fatty acid oxidation. The present finding that HSL phosphorylation was lower in recovery from exercise suggest that adipose tissue limits lipolysis in the hours after exercise to regain resting levels of plasma NEFA. Remarkably, iWAT HSL^Ser660^ phosphorylation was unchanged after exercise in IL-6 MKO mice and in eWAT significantly higher, indicating that IL-6 MKO mice was unable to regulate lipolysis during recovery from exercise. This may suggest that the lack of skeletal muscle IL-6 was associated with loss of lipolytic control both at rest and during recovery from acute exercise. A recent study suggested that increased lipolysis can lead to higher P38 phosphorylation in adipose tissue [32]. The present findings that P38^Thr180/Tyr182^ phosphorylation was higher in IL-6 MKO than Control mice in both iWAT and eWAT 6h after exercise are in agreement with these observations and may indicate that knockout of skeletal muscle IL-6 led to intracellular stress in adipose tissue. It is noteworthy that several studies suggest that increased P38-phosphorylation in adipose tissue can lead to insulin resistance [32, 33]. Thus, it is possible that the elevated p38 phosphorylation in IL-6 MKO mice may have resulted in reduced insulin sensitivity in adipose tissue. Previous findings have shown that insulin increases GLUT4 expression in adipose tissue [34, 35] and the present findings indicate that plasma insulin increased similarly in Control and IL-6 MKO mice 10h after exercise. Therefore, it may be speculated that reduced insulin signaling in adipose tissue 10h after exercise accounts for the observed loss of the exercise-induced increase in iWAT GLUT4 protein content late in recovery from exercise in IL-6 MKO mice. These findings suggest that secretion of skeletal muscle IL-6 during exercise may be important for reducing lipolysis and stress kinase signaling in iWAT during recovery from exercise.

IL-6 has been shown to be important for the regulation of glucose and fat metabolism in several studies [9, 10, 13, 14]. The observation that lack of skeletal muscle IL-6 did not alter ACC or AMPK phosphorylation in skeletal muscle 6 and 10h after exercise, suggests that skeletal muscle fat oxidation was unaltered, which is not in accordance with previous studies indicating that IL-6 increases fat oxidation [17, 31]. Furthermore, the increased skeletal muscle PDHa activity in the IL-6 MKO mice both at 6h after exercise and in non-exercised mice indicates that carbohydrate oxidation was higher when muscle IL-6 was lacking at these time points. On the other hand, the lack of genotype difference in PDHa activity at 10h of recovery may suggest that there could be a circadian influence on the IL-6-mediated regulation of PDHa activity. This possibility is in accordance with the previous observations that PDHa activity indeed oscillates in a circadian manner [36, 37]. The current findings, indicating that muscle IL-6 reduces skeletal muscle PDHa activity and hence likely carbohydrate oxidation, are in contrast to previous observations showing that IL-6 infusion in humans increased skeletal muscle glucose uptake [10] and increased GLUT4 translocation in C2C12 myotubes [38]. However, the present observation is in agreement with the previous finding that lack of skeletal muscle IL-6 increased PDHa activity in non-exercised mice and during exercise and resulted in higher respiratory exchange ratio (RER) during prolonged exercise [12]. Similarly, the previous finding that injection of IL-6 decreased PDHa activity in fed mice [11] further supports the current observation. Together this may suggest that skeletal muscle IL-6 is an important negative regulator of carbohydrate oxidation at rest and after exercise, but also underlines that IL-6 elicits pleiotropic effects, which may vary with changes in the metabolic conditions.

The higher plasma lactate in IL-6 MKO than Control mice observed in non-exercised mice and 6h after exercise may seem contradictory to the finding that PDHa activity was higher and therefore potentially associated with reduced lactate production in skeletal muscle of IL-6 MKO mice. On the other hand, it may be that the higher PDHa activity reflects an elevated glycolytic flux and thus increased pyruvate and lactate production within muscle tissue of IL-6 MKO mice. Alternatively, hepatic lactate uptake may have been reduced when muscle IL-6 was lacking. However, the higher PEPCK protein content 10h after exercise in both genotypes, without changes in PEPCK mRNA and G6Pase mRNA or protein content, does not support that IL-6 MKO mice had altered gluconeogenic capacity, which is in line with previous studies [6, 39]. Thus, the increased plasma lactate concentration in IL-6 MKO than in Control mice may be due to reduction in hepatic lactate uptake, but the similarly reduced liver lactate 6h post exercise in both genotypes seems to indicate that the higher plasma lactate is because of higher peripheral lactate production.

It remains unclear whether the effects of muscle IL-6 on glucose metabolism are directly exerted on skeletal muscle metabolism or via IL-6 mediated effects in other tissues or perhaps via other mediators. Hence, IL-6 has been shown to increase the production and release of Glucagon like peptide 1 (GLP-1) during exercise [40], suggesting that increased levels of IL-6 during exercise may be important for the regulation of GLP-1 with concomitant effects on insulin secretion after exercise. Thus, it may be suggested that muscle IL-6 plays a dual role by regulating substrate utilization in skeletal muscle and improving insulin secretion after acute exercise. However, the present observation that plasma insulin was not different between genotypes does not support that muscle IL-6 affects post exercise insulin secretion.

It has previously been reported that lack of skeletal muscle IL-6 did not alter muscle STAT3 phosphorylation or plasma IL-6 concentration during exercise [12]. In the present study, the plasma concentration of IL-6 did not differ significantly between genotypes, which may be due to the considerable variation in plasma IL-6 observed or that other tissues such as adipose tissue or immune cells may have compensated [41]. Several isoforms of IL-6 with different properties emanating from different tissues have also been proposed. As the current methodologies stand and with the knowledge about the biology of IL-6 it is not possible to distinguish between IL-6 emanating from differentsources. Thus, it is possible that redundant secretion from other tissues will have reduced the observed effects of skeletal muscle IL-6 knockout in the current experimental setup. Of notice is, however, that lack of skeletal muscle IL-6 nonetheless did result in changes in metabolic regulation, mRNA and protein levels in skeletal muscle and adipose tissue both in non-exercised mice and during recovery from prolonged exercise. The observation that these effects were not associated with detectable alterations in canonical IL-6 signaling in skeletal muscle and adipose tissue, but that hepatic SOCS3 mRNA increased in Control, but not in IL-6 MKO mice 10h after exercise suggests that lack of skeletal muscle IL-6 influenced canonical IL-6 signaling in recovery from exercise only in the liver. Thus, it seems possible that the effect of skeletal muscle IL-6 knockout on skeletal muscle and adipose tissues were elicited through pathways other than canonical IL-6 signaling.

In conclusion, the present findings may suggest that skeletal muscle IL-6 plays a role in the regulation of substrate utilization in skeletal muscle as well as the regulation of lipolysis, GLUT4 expression and stress kinase signaling, in adipose tissue late during recovery from exercise. In addition, skeletal muscle IL-6 does not seem to be required for regulation of the gluconeogenic capacity late in recovery from exercise.
